# Unique haplotypes in ant-attended aphids and widespread haplotypes in non-attended aphids

**DOI:** 10.1002/ece3.348

**Published:** 2012-08-13

**Authors:** Izumi Yao, Takashi Kanbe

**Affiliations:** Systematic Entomology, Department of Ecology and Systematics, Graduate School of Agriculture, Hokkaido UniversitySapporo, 060-8589, Japan

**Keywords:** Aphid-ant mutualisms, COI, haplotype diversity, outgroup heteroduplex, *Quercus dentata*, *Tuberculatus* aphids

## Abstract

Aphid species within the genus *Tuberculatus* Mordvilko (Hemiptera: Aphididae) exhibit a variety of interactions with ants, ranging from close associations to non-attendance. A previous study indicated that despite wing possession, ant-attended *Tuberculatus* species exhibited low dispersal rates compared with non-attended species. This study examined if presence or absence of mutualistic interactions and habitat continuity of host plants affected intraspecific genetic diversity and genetic differentiation in mitochondrial DNA cytochrome oxidase I (COI) sequences. Sympatric ant-attended *Tuberculatus quercicola* (Matsumura) (Hemiptera: Aphididae) and non-attended *Tuberculatus paiki* Hille Ris Lambers (Hemiptera: Aphididae) were collected from the daimyo oak *Quercus dentata* Thunberg (Fagales: Fagaceae) in Japan and examined for haplotype variability. Seventeen haplotypes were identified in 568 *T. quercicola* individuals representing 23 populations and seven haplotypes in 425 *T. paiki* representing 19 populations. Haplotype diversity, which indicates the mean number of differences between all pairs of haplotypes in the sample, and nucleotide diversity were higher in *T. quercicola* than *T. paiki*. Analysis of molecular variance (AMOVA) showed higher genetic differentiation among populations within groups of *T. quercicola* (39.8%) than *T. paiki* (22.6%). The effects of attendant ant species on genetic differentiation in *T. quercicola* were not distinguishable from geographic factors. Despite low dispersal rates, host plant habitat continuity might facilitate widespread dispersal of a *T. quercicola* haplotype in Hokkaido. These results suggested that following *T. quercicola* colonization, gene flow among populations was limited, resulting in genetic drift within populations. However, frequent *T. paiki* dispersal is clearly evident by low genetic differentiation among populations within groups, resulting in lower haplotype diversity.

## Introduction

The aphid genus *Tuberculatus* Mordvilko (Hemiptera: Aphididae) feeds primarily on Fagaceae, does not alternate host plants during its life history, and exhibits various interactions with ants, ranging from non-attendance to strong associations. *Tuberculatus* species exhibit cyclical parthenogenesis; During the summer months, *Tuberculatus* nymphs develop into winged (alate) viviparous females, regardless of host plant nutritional quality, aphid colony density, or ant attendance. In autumn, alate males and apterous oviparous females appear. After mating, oviparous females move from the leaves to the branches to deposit eggs.

Attending ants often have negative impacts on aphids, including decreased body size and/or embryo number due to the costs of increased honeydew production (Stadler and Dixon 1998; Yao et al. 2000; Yao and Akimoto 2001, 2002) and suppression of colony development (Katayama and Suzuki 2002). Furthermore, attending ants inhibit aphid dispersal. Ant mandibular secretions can inhibit alata development (Kleinjan and Mittler 1975) and ant semiochemicals can reduce the walking activity of apterous aphids (Oliver et al. 2007). These examples suggest that the dispersal range of ant-attended aphids is limited to relatively small fragmented areas with limited gene flow between them.

**Figure 1:**
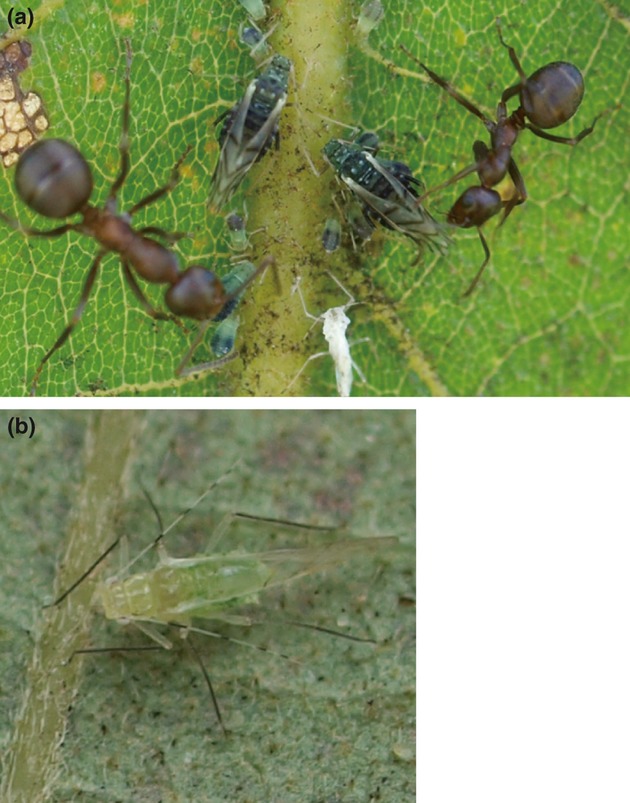
Ant-attended aphid (a) *Tuberculatus quercicola* and non-attended aphid (b) *Tuberculatus paiki*.

In a previous field study using flight intercept traps and weekly observations, results showed that two strongly ant-attended species, *Tuberculatus quercicola* (Matsumura) (Hemiptera: Aphididae) (Fig. 1a) and an undescribed *Tuberculatus* sp. A, exhibited extremely low dispersal levels compared with two non-attended species, *Tuberculatus japonicus* Higuchi (Hemiptera: Aphididae) and *Tuberculatus paiki* Hille Ris Lambers (Hemiptera: Aphididae) (Fig. 1b): the total numbers of winged individuals trapped and observed (trapped/observed) in trees throughout all seasons were eight/1342 for *T. quercicola*, two/194 for *T*. sp. A, 52/200 for *T. japonicus*, and 137/1315 for *T. paiki* (Yao 2010). Moreover, isolation by distance is not found in *T. quercicola* populations at microgeographic scales, where the mean distance between host trees is 240 m (Yao and Akimoto 2009). These studies demonstrated that gene flow in ant-attended *Tuberculatus* species was limited to within a small range.

Genetic diversity is influenced by many factors, including animal and seed dispersal capacity, and habitat continuity. The daimyo oak *Quercus dentata* Thunberg (Fagales: Fagaceae) is a deciduous broad-leaved tree, which is host to *T. quercicola* and *T. paiki*. The species is native to Japan, Korea, and China, and its habitat ranges from the seacoast to highland regions. Pollen fossil records indicate that daimyo oak was widely distributed along the Sea of Japan (Yasuda and Miyoshi 1998), however, *Q. dentata* woods along the seacoast were cut for fuel replaced by the Japanese Black Pine *Pinus thunbergii* Parlatore (Pinales: Pinaceae) to serve as a windbreak, or lost due to the development of more desirable landscapes. Such an anthropogenic fragmentation of *Q. dentata* populations would have significant impact on the population genetic structure and intraspecific phylogenetic divergence of *T. quercicola*, but not *T. paiki*.

Organellar genomes are well accepted as appropriate in estimating population history, and information regarding genealogical relationships between and within samples can typically be obtained from the appropriate genes. Furthermore, between and within taxa comparisons are rapidly and easily performed. In this study, genetic structure of *Tuberculatus* aphids was examined using mitochondrial DNA cytochrome oxidase I (COI) sequences. The two species are known to exhibit contrasting dispersal patterns, therefore unique haplotypes were expected in *T. quercicola* populations, and widespread haplotypes in *T. paiki* populations.

This study examined whether the presence or absence of mutualistic interactions affected intraspecific genetic diversity in mitochondrial COI sequences, and focused on ant-attended *T. quercicola* and non-attended *T. paiki* species collected from an approximately 1800 km length in Japan. Population histories of the two species are discussed in terms of dispersal capacity and habitat continuity of host plants.

## Materials and Methods

### Geographic groups

**Figure 2:**
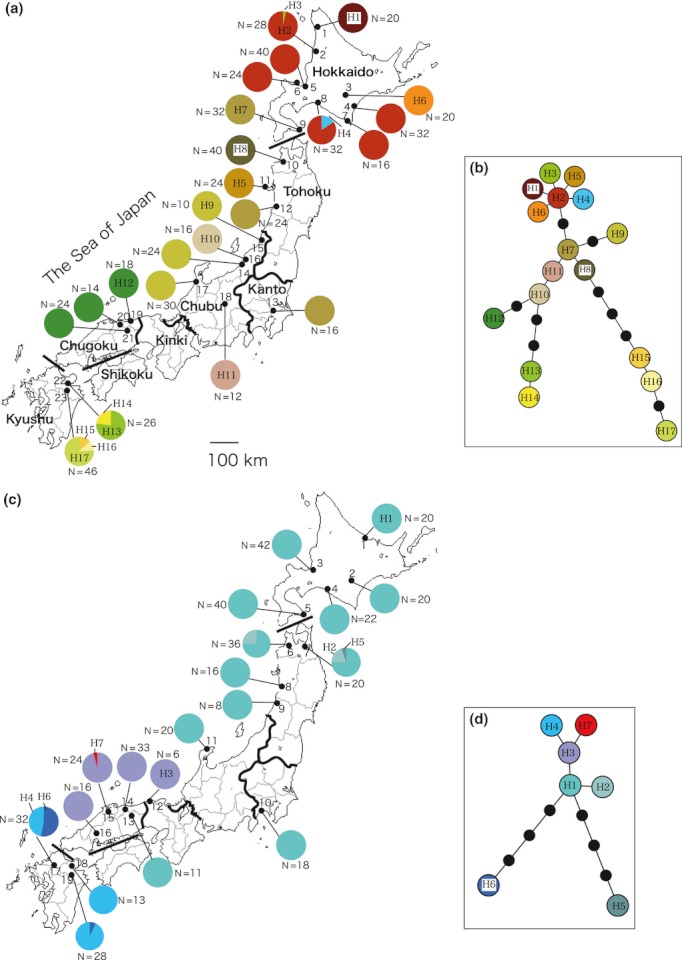
Haplotype distribution of (a) *Tuberculatus quercicola* and (c) *Tuberculatus paiki*. Number in the pie chart and N indicate haplotype code and sample size, respectively. Numbers in the map designate collection sites shown in Table 1. Bold lines indicate group boundaries. Diagrams at right show relationships between species haplotypes (b) *T. quercicola* and (d) *T. paiki*.

The regions of Japan were divided into eight major regional geographic groups: Hokkaido, Tohoku, Kanto, Chubu, Kinki, Chugoku, Shikoku, and Kyushu. Due to the absence of available samples, the Shikoku region was excluded from analyses and the Kinki region for *T. quercicola*. The seven regions were assigned to three islands divided by the strait: Hokkaido, Honshu, and Kyushu (Table 1; Fig. 2).

**Table 1:** Collection data for *Tuberculatus quercicola* and *Tuberculatus paiki*. *N* indicates the number of aphids used in genotyping

		*T. quercicola*					*T. paiki*			
				
Island^a^	Region Group	Population		*N*	Haplotype	Ants^b^	Population		*N*	Haplotype
Hokkaido	Hokkaido	1	Teshio	20	H1	Fy	1	Saroma	20	H1
		2	Tomamae	28	H2, 3	Fy, Fj	2	Obihiro	20	H1
		3	Obihiro	20	H6	Lj	3	Ishikari	42	H1
		4	Churui	32	H2	Fy	4	Mukawa	22	H1
		5	Ishikari	40	H2	Fy	5	Esan	40	H1
		6	Oshoro	24	H2	Fy				
		7	Erimo	16	H2	Ff, Lj				
		8	Mukawa	32	H2, 4	Lj				
		9	Esan	32	H7	Lj				
Honshu	Tohoku	10	Syariki	40	H8	Lj	6	Syariki	36	H1, 2
		11	Nyudozaki	24	H5	Pp	7	Shichinohe	20	H1, 2, 5
		12	Kisakata	24	H7	Lj	8	Iwaki	16	H1
							9	Misaki	8	H1
	Kanto	13	Kashiwa	16	H7	Ls	10	Hadano	18	H1
	Chubu	14	Kashiwazaki	24	H9	Pp, Lj	11	Wajima	20	H1
		15	Iwagasaki	10	H9	Pp				
		16	Iwamuro	16	H10	Ct				
		17	Oshimizu	30	H9	Lj				
		18	Matsumoto	12	H11	Lj				
	Kinki	-	-	-	-	-	12	Kasumi	6	H3
	Chugoku	19	Aoya	18	H12	Pp	13	Kawakamison	11	H1
		20	Daisen	14	H12	Lj	14	Daisen	33	H3
		21	Kawakamison	24	H12	Lj	15	Unnan	24	H3, 7
							16	Geihoku	16	H3
Kyushu	Kyushu	22	Yufudake	26	H13, 14	Cj	17	Sasaguri	32	H4, 6
		23	Kokonoe	46	H15, 16, 17	Lj	18	Yufudake	13	H4
							19	Kokonoe	28	H4, 6
			Total	568					425	

1 Major island in Japan divided by a strait.

2 Attendant ant species.

Cj, *Camponotus japonicus*; Cr, *Crematogaster teranishii*; Ff, *Formica fukaii*; Fj, *Formica japonica*; Fy, *Formica yessensis*; Lj, *Lasius japonicus*; Ls, *Lasius sakagamii*; Pp, *Pristomyrmex punctatus*.

### Sample collection

Totals of 568 *T. quercicola* and 425 *T. paiki* were collected from 23 and 19 populations, respectively, on *Q. dentata* from the years 2005 to 2011. Sample collection was conducted on viviparous females (third to fourth instars or winged adults), which appeared from late May to mid September. Colonies of *T. quercicola* were attended by eight ant species, including *Camponotus japonicus* Mayer (Hymenoptera: Formicinae), *Crematogaster teranishii* Santschi (Hymenoptera: Myrmicinae), *Formica fukaii* Wheeler (Hymenoptera: Formicinae)*, Formica japonica* Motschoulsky (Hymenoptera: Formicinae), *Formica yessensis* Forel (Hymenoptera: Formicinae), *Lasius japonicus* Santschi (Hymenoptera: Formicinae), *Lasius sakagamii* Yamauchi and Hayashida (Hymenoptera: Formicinae), and *Pristomyrmex punctatus* Smith (Hymenoptera: Myrmicinae) (Table 1). Multiple aphid clone collection was avoided by sampling a single aphid from each sampled leaf. All aphids and ants were preserved in vials containing with 99.5% ethanol.

### DNA extraction and screening of haplotypes by outgroup heteroduplex analysis

Total DNA was extracted from the entire aphid following the Chelex procedure (Walsh et al. 1991). Haplotype polymorphisms were screened throughout a population sample using outgroup heteroduplex analysis (Campbell et al. 1995). This technique forms heteroduplexes by mixing sequences of a target and a reference (outgroup) species. Heteroduplex products, in which two strands with mismatched bases form bulges at the homologous sites during annealing (White et al. 1992) have unique electrophoretic mobility depending on their configuration in non-denaturing polyacrylamide gels (Nagamine et al. 1989). Heteroduplex products consisting of a higher ratio of mismatched bases to entire bases in a sequence (up to 500 bp) produce more distinct electrophoretic mobility, so that anterior and posterior halves of interesting regions of mitochondrial COI were amplified separately. Primer sets, C1-J-1718 (5′-GGA GGA TTT GGA AAT TGA TTA GTT CC-3′) (Simon et al. 1994) + R2191 (5′-CCC GGT AAA ATT AAA ATA TAA ACT TC-3′) and TQ-INT-F (5′-CAA GCA CAT TTA TTC TGA TTT TTT GG-3′) + TQ-INT-R (5′-GGG AAT CAG TGA ATG AAT CTT GC-3′) were used to amplify the two partial COI regions. Polymerase chain reaction (PCR) was performed in 10-*μ*L volumes, which included 1 *μ*L of 10× PCR buffer (Takara-Bio, Otsu, Japan), 0.8 *μ*L of dNTP mixture (2.5 mM of each), 0.5 *μ*L of 2 pM of each primer, 10 ng/*μ*L of genomic DNA, and 0.025 units of Ex-Taq DNA polymerase (Takara-Bio). The reaction cycle parameters were as follows: 94°C for 3 min; 30 cycles of 94°C for 30 sec, 45°C for 20 sec, and 65°C for 90 sec. For heteroduplex formation, 2 *μ*L of *T. quercicola* PCR products was mixed with 2 *μ*L of *T. paiki* PCR product amplified from a single aphid using the same above conditions, and standard loading buffer. The mixture was heated to 94°C for 5 min, and slowly cooled to room temperature. One microliter of mixture was electrophoresed on 13-cm non-denaturing gels consisting of 10% acrylamide (36:1 acrylamide:bisacrylamide) in 1× TBE buffer at 150V for approximately 5 h. Gels were stained for 20 min in 1× TBE containing 0.5 μg/ml ethidium bromide, and examined using a ultraviolet transilluminator.

### Sequencing

Each different haplotype identified in a population was sequenced. All PCR reactions were performed in 20-*μ*L volumes, which included 2 *μ*L of 10× PCR buffer (Takara-Bio), 1.6 *μ*L of dNTP mixture (2.5 mM of each), 1 *μ*L of 2 pM of each primer, 2*μ*L of 10 ng/*μ*L of genomic DNA, and 0.5 units of Ex-Taq DNA polymerase (Takara-Bio). The reaction cycle parameters were as follows: 94°C for 3 min; 30 cycles of 94°C for 30 sec, 45°C for 20 sec, and 65°C for 90 sec. For each fragment, the entire PCR product was purified using the QIAquick PCR purification kit (QIAGEN, Tokyo, Japan). A 5-*μ*L sequencing reaction volume was used and consisted of 2 *μ*L of Quick Start Mix (Bechman Coulter, Tokyo, Japan), 0.5 *μ*L of 10 pM forward or reverse primers, and 2.5 *μ*L of 10 ng/*μ*L template DNA. The reaction cycle parameters were as follows: 33 cycles of 94°C for 30 sec, 50°C for 15 sec, and 65°C for 90 sec. DNA sequencing was performed using CEQ2000XL DNA Analysis System (Bechman Coulter). Totals of 875 and 862 bp were aligned for *T. quercicola* and *T. paiki*, respectively. Alignment was conducted manually using MacClade 4.08 (Maddison and Maddison 2005). Sequences of COI were deposited in the DNA Data Bank of Japan under accession numbers AB679242 – AB679258 for *T. quercicola* haplotypes 1 to 17 and AB679259 – AB679265 for *T. paiki* haplotypes 1 to 7.

### Genetic diversity and population genetics analysis

Haplotype diversity, haplotype mean pairwise distance, nucleotide diversity (*π*), and mutation rates (*θ* (*S*)) were calculated to examine species genetic diversity. Haplotype diversity is defined as the mean number of differences between all pairs of haplotypes. Mutation rate is an estimate of the scaled mutation rate determined from the number of segregating sites (*S*) in a sample of DNA sequences. A segregating site is any of the total number of nucleotide sites that maintain two or more nucleotides within population (Hamilton 2009). Gene genealogies were estimated by constructing a haplotype network using HapStar (Teacher and Griffiths 2011). Population demographic patterns were estimated by calculating mismatch distributions and Tajima's *D* (Tajima 1989). Mismatch distributions from populations that had experienced a constant *Ne* (effective population size) over time tend to exhibit a bimodal distribution, where two clusters of values in the mismatch distribution are evident (Hamilton 2009). A L-shaped distribution indicates recent population growth, or balancing selection. In contiguous populations, a unimodal distribution will appear in demographic expansion (Excoffier 2004). Tajima's *D* statistics were subsequently applied to calculate selective neutrality of haplotype. The statistics use the nucleotide diversity (*π*) and the number of segregating sites (*S*) observed in a sample of DNA sequences to make two estimates of the scaled mutation rate, *θ* (*S*) and *θ* (*π*). *D* < 0 (*θ* (*π*) < *θ* (*S*)) indicates populations that had experienced rapidly growing. *D* > 0 (*θ* (*π*) > *θ* (*S*)) indicates populations that had experienced recent bottleneck. Genetic differentiation among populations was assessed using an analysis of molecular variance (AMOVA). Populations analyzed under AMOVA were a priori divided into six and seven of the eight major regional geographic groups for *T. quercicola* and *T. paiki*, respectively. An additional AMOVA was performed following a subdivision according to *T. quercicola* attendant ant species. Population genetics data were analyzed using Arlequin (Schneider et al. 2000). The extent of range expansion in both species was estimated by applying mismatch distributions based on coalescent simulations (Excoffier 2004) to haplotypes that were pooled in three areas: Hokkaido, Honshu (main island), and Kyushu.

### Phylogenetic analysis

Bayesian analysis was conducted using MrBayes ver. 3.1.2 (Ronquist and Huelsenbeck 2003), and the GTR + I model selected by MrModeltest ver 2 (Nylander 2004). The number of generations, sample frequency, and burn-in were set to 10^6^, 100, and 500, respectively.

## Results

### Genetic diversity and population genetics analysis

**Figure 3:**
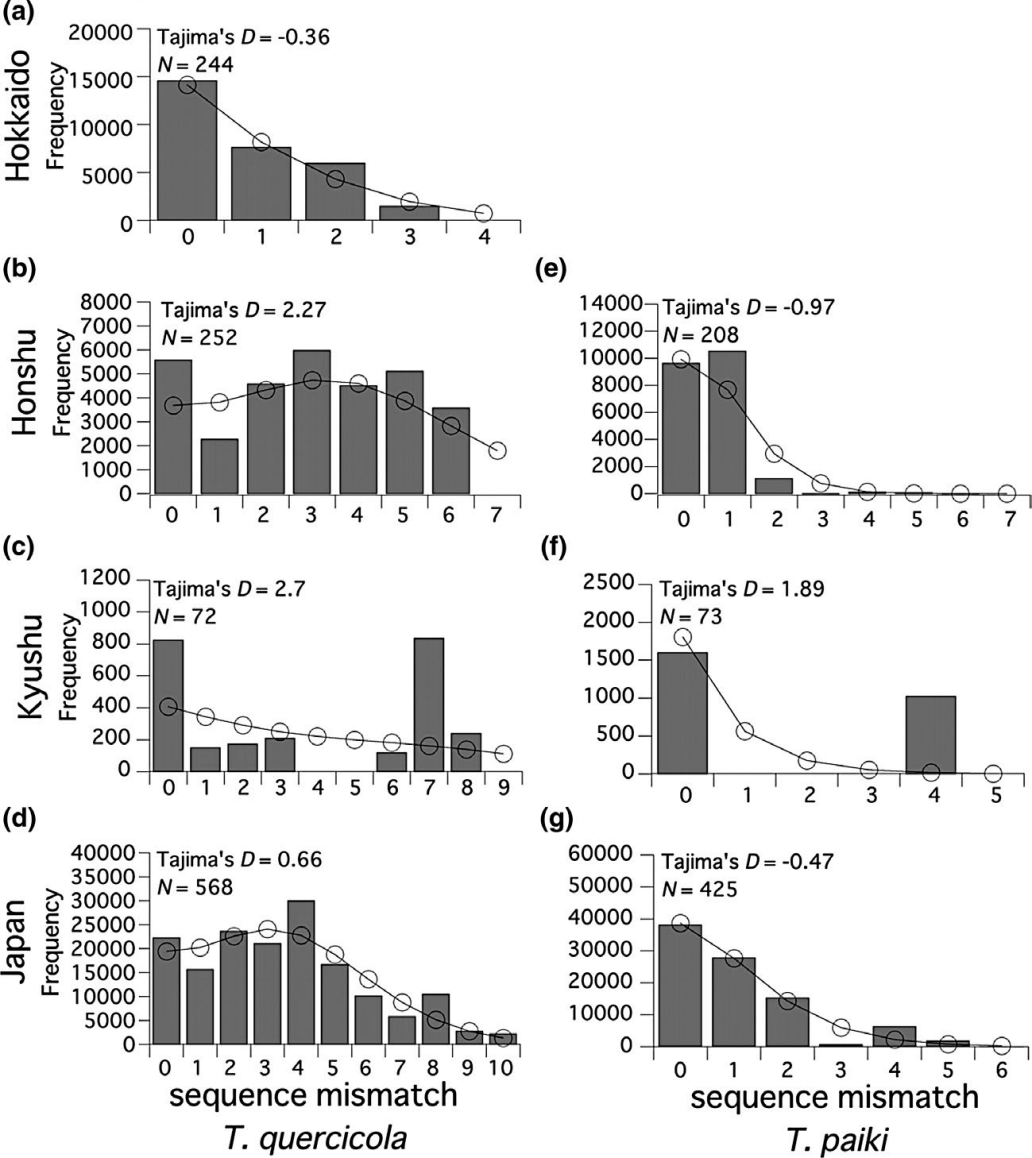
Mismatch distributions with Tajima's *D* values for *Tuberculatus quercicola* populations in (a) Hokkaido, (b) Honshu, (c) Kyushu, and (d) Japan; and for *Tuberculatus paiki* populations in (e) Honshu, (f) Kyushu, and (g) Japan. Gray bars and open circles indicate observed mismatch and model frequency.

Seventeen *T. quercicola* and seven *T. paiki* haplotypes were detected among all samples (Fig. 2a and c). Haplotype diversity and nucleotide diversity were higher in *T. quercicola* than *T. paiki* (Table 2). The following populations included more than one haplotype: *T. quercicola* populations 2, 8, 22, and 23, and *T. paiki* populations 6, 7, 15, 17, and 19. Twelve of 19 *T. paiki* populations possessed haplotype 1. Nine and six missing haplotypes were detected in *T. quercicola* and *T. paiki* networks, respectively (Fig. 2b and d). Mismatch distribution analysis showed that *T. quercicola* had three graph shapes: left-skewed, unimodal, and bimodal shapes appeared in Hokkaido, Honshu, and Kyushu populations, respectively (Fig. 3a–c). Tajima's *D* for *T. quercicola* showed positive values in Honshu (2.27), Kyushu (2.7), and all samples from Japan (0.66), but negative value in Hokkaido populations (−0.36). Mismatch distribution of *T. paiki* showed left-skewed and bimodal shapes in Honshu and Kyushu populations, respectively (Fig. 3e and f). Mismatch distribution analysis was not conducted on Hokkaido *T. paiki* populations, which had a single haplotype. Tajima's *D* for *T. paiki* showed positive value in Kyushu (1.89) and negative values in Honshu (−0.97) and all samples from Japan (−0.47). Mismatch distribution of all samples from Japan exhibited unimodal and positively skewed distributions in *T. quercicola* (Fig. 3d) and *T. paiki* (Fig. 3g) populations, respectively.

**Table 2:** Genetic diversity of *Tuberculatus quercicola* and *Tuberculatus paiki*. Haplotype diversity indicates an estimation and its sampling variance. Mean number of pairwise differences indicates mean number of differences between all pairs of haplotypes in the sample and total variance. Nucleotide diversity (π) indicates the probability that two randomly chosen homologous nucleotides are different and its variance. Mutation rate (*θ* (*S*)) indicates an estimate of the scaled mutation rate determined from the number of segregating sites in a sample of DNA sequences

	No. haplotypes	No. polymorphic sites	Haplotype diversity	Mean number of pairwise differences	Nucleotide diversity (π)	Mutation rate (*θ* (*S*))
*T. quercicola*	17	19	0.86±0.01	3.5±1.79	0.004±0.0023	2.75±0.8
*T. paiki*	7	9	0.58±0.02	1.05±0.7	0.0012±0.0009	1.36±0.51

For both species, AMOVA indicated the genetic variance among groups accounted for more than 50% of the total variation (i.e., 57.4% in *T. quercicola* and 54.2% in *T. paiki*). However, genetic differentiation among populations within groups was higher in *T. quercicola* (39.8%) than in *T. paiki* (22.6%). AMOVA for subdivision by attendant ant species exhibited high genetic variance within species, responsible for 61% of the total variation (Table 3).

**Table 3:** Analysis of molecular variance for *Tuberculatus quercicola* and *Tuberculatus paiki*

Source of variation	df	Sum of squares	Variance of components	Percentage of variation	Fixation indices	*P*
Geographic subdivision for *T. quercicola*						
Among groups	5	614.71	1.21	57.44	FCT = 0.57436	0.0001
Among populations within groups	17	345.94	0.84	39.8	FSC = 0.93496	0.0001
Within populations	545	31.76	0.06	2.77	FST = 0.97232	0.0001
Total	567	992.40	2.10			
Ant species subdivision for *T. quercicola*						
Among ant genus	4	287.68	0.65	32.03	FCT = 0.32030	0.00196
Among ant species within ant genus	2	10.90	0.14	7.03	FSC = 0.10341	0.0001
Within ant species	561	693.83	1.24	60.94	FST = 0.39059	0.0001
Total	567	992.40	2.03			
Geographic subdivision for *T. paiki*						
Among groups	6	121.32	0.31	54.16	FCT = 0.54159	0.001
Among populations within groups	12	37.31	0.13	22.55	FSC = 0.49202	0.0001
Within populations	406	54.97	0.14	23.29	FST = 0.76714	0.0001
Total	424	213.60	0.58			

### Phylogenetic analysis

**Figure 4:**
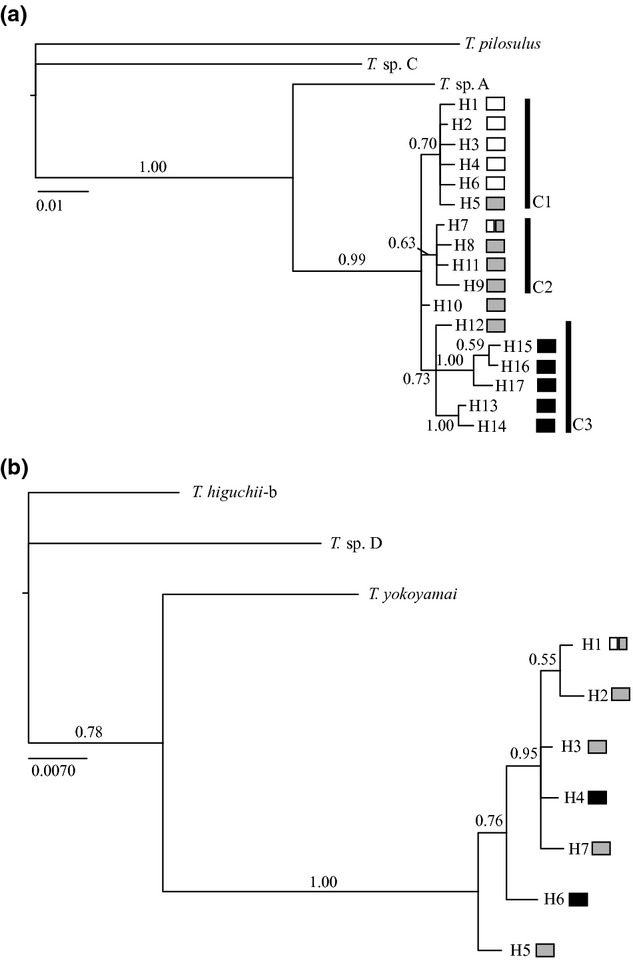
Phylogenetic trees derived from haplotypes in (a) *Tuberculatus quercicola* and (b) *Tuberculatus paiki* obtained from the Bayesian analysis method. Probabilities >0.5 are shown below or near the branches. Bold lines in (a) indicate clades (C1, C2, and C3) that form monophyletic groups. Rectangles indicate the geographic areas where each haplotype was collected: white, gray, and black correspond to Hokkaido, Honshu, and Kyushu, respectively.

Phylogenetic analysis showed three *T. quercicola* clades in the Bayesian tree (Fig. 4a). The first clade included all but haplotype 7 found in the Hokkaido group. The second clade was comprised of haplotypes detected in Hokkaido, Tohoku, and Kanto groups. Haplotype 5 from individuals of population 11 was allied with clade 1. The third clade included haplotypes representing the Chugoku and Kyushu groups. In *T. quercicola*, different haplotypes from the same population were closely positioned on the tree, particularly the Kyushu haplotypes, where monophyletic groups were formed with high probability of group support. In contrast, the *T. paiki* haplotypes did not form distinctive clades representing geographic groups (Fig. 4b). Haplotypes 1 and 2 in the Tohoku group formed a monophyletic group; however, haplotypes 4 and 6 in the Kyushu group were paraphyletic.

## Discussion

This study revealed that *T. quercicola* populations possessed 2.4 times more unique geographically structured haplotypes relative to *T. paiki*, which exhibited a widely distributed haplotype (haplotype 1) throughout several groups. One of the primary factors affecting genetic diversity is the level of gene flow among populations. AMOVA results for *T. quercicola* indicated the variation in the second and third hierarchical levels (i.e*.*, among populations within groups, and within populations) accounted for approximately 40% and 3% of the total genetic variation, respectively, indicating limited gene flow within relatively small geographic areas. The low *T. quercicola* dispersal rates may be associated with physical flight difficulties and fitness benefits from ant attendance. A comparative study on flight muscle in *T. quercicola* and *T. paiki* showed that flight muscle development was significantly lower in *T. quercicola* than *T. paiki* (Yao and Katagiri 2011). Yao et al. (2000) found that approximately half of *T. quercicola* colonies suffered mortality within a month due to predation pressures under experimental conditions that excluded attending ants. Low dispersal rates due to the physical constraints, and beneficial services from ant protection would result in long periods of geographically fixed colonization, and presumably the accumulation of mutations, ultimately resulting in unique haplotypes origins in *T. quercicola*. Our results detected double the mutation rate (*θ* (*S*)), and three times the mean number of pairwise differences and nucleotide diversity in *T. quercicola* compared with *T. paiki*, indicating that mutation following colonization by *T. quercicola* in each newly colonized population might lead to genetic differentiation. Mismatch distribution and positive Tajima'*D* of all samples from Japan indicated that *T. quercicola* populations underwent recent bottleneck. Limited dispersal by ant attendance makes aphid colonies small populations, so that bottleneck would have been more effective in such small populations.

The processes described above serve to describe the historical factors resulting in multiple *T. quercicola* Kyushu group haplotypes. Definitive phylogenetic relationships were observed among Kyushu group haplotypes in populations 22 and 23. Phylogenetic analysis showed a monophyletic group derived of clade 3 supported by a high bootstrap value. Furthermore, the bimodal pattern in mismatch distribution (Fig. 3c) clearly indicated that the two populations shared a common ancestral lineage, and were therefore separated for a longer period of time relative to other populations. These results suggested that ancestral and descendent haplotypes were maintained in the same population due to low dispersal, resulting in mixed haplotypes in *T. quercicola* populations.

Despite presumed low dispersal rates, widely distributed haplotype 2 was detected in the *T. quercicola* Hokkaido group. Mismatch distribution (Fig. 3a) and negative Tajima's *D* value also suggested recent and rapid expansion of haplotype 2. The lack of congruence could result from contiguous habitat effects, and low mitochondrial marker resolution. *Q. dentata* exhibits a widespread distribution in northern Japan, particularly from northern Tohoku to Hokkaido, where severe winter seasonal wind prevents occurrence of other deciduous tree species (Tamura et al. 1999). The climatic and ecological conditions supporting *Q. dentata* as the single overstory deciduous species might facilitate widespread haplotype 2 distribution in Hokkaido. However, Yao (2010) showed genetic differentiation among 11 *T. quercicola* Hokkaido populations using microsatellite markers, suggesting that attending ants limit current gene flow in *T. quercicola* populations.

AMOVA analysis following subdivision according to attendant ant species indicated the percentage of variation in the third hierarchy (i.e., within ant species) accounted for 61% of the total genetic variation. However, ant species habitat and geographic subdivision (analyzed in the first AMOVA) were not independent, suggesting *T. quercicola* genetic structure may derive from confounding factors including geographic distribution and attendant ant species.

In contrast to *T. quercicola*, frequent dispersal of *T. paiki* is responsible for low genetic differentiation among populations within groups, resulting in lower haplotype diversity. Mismatch distribution and negative Tajima's *D* of all samples of *T. paiki* from Japan also indicates recent expansion. Haplotype 1 was widely distributed from the Chubu to Tohoku areas and in Hokkaido across the Tsugaru Strait, where the narrowest point between the Tohoku and Hokkaido is approximately 19 km. The mismatch distribution (Fig. 3e) and negative Tajima's *D* value for the Honshu populations indicate a history of very rapid population growth in the recent past. Aphids are known to fly using fast-moving airstreams at high altitude (relative to insect flight). *Tuberculatus annulatus* (Hartig) (Hemiptera: Aphididae), a non-attended *Tuberculatus* aphid, was caught by aerial netting at ∼200 m above land (Chapman et al. 2004). Therefore, dispersal of haplotype 1 between Hokkaido and Tohoku is a plausible hypothesis to explain distribution of this haplotype.

Haplotype 3 detected in the Kinki and Chugoku groups, and haplotypes 4 and 6 in the Kyushu group exhibited discrete geographic distribution patterns in *T. paiki*. *Quercus dentata* woods have shown a rapid and distinct decline in the Chugoku area coastline, and remain locally in an artificial forest (population 17) and highlands surrounded by mountains (populations 18 and 19). The disruptive effects of these geographic boundaries can serve as a barrier to gene flow, and subsequently the observed haplotype distribution.

The three *T. paiki* haplotypes (H4, H5, and H6) exhibited little phylogenetic relationship with other haplotypes in each population. This was further clarified from the haplotype network. Missing haplotypes between H4 and H6, and between H5 and H1 or H2 indicated that phylogenetically distant haplotypes entered a population by chance likely due to widespread dispersal. Considering the phylogenetic relationships between H4 and H6, and the mono-haplotype in population 18, H6 is likely to have migrated from outside Japan.

This study clearly revealed contrasting patterns of genetic diversity between ant-attended *T. quercicola* and non-attended *T. paiki*. However, recent interspecific comparative studies have been developed in the framework of phylogenetic relationships (Felsenstein 1985), indicating that the results of this study should be treated with caution. As *T. quercicola* and *T. paiki* are not sister species (Yao 2011), it is possible that the differences in the genetic structure between the two species may be attributed to the relative phylogenetic position rather than mutualistic interactions with ants. However, Yao (2011) used phylogenetic comparative methods to demonstrate the parallel evolution of higher wing loading with ant attendance. Ultimately, extensive studies on the morphological, physiological, reproductive, and phylogenetic relationships of other *Tuberculatus* species would contribute toward elucidating the evolutionary processes in aphid–ant interactions.
